# Lessons learned from an integrated mapping and ablation catheter

**DOI:** 10.1016/j.hroo.2026.02.032

**Published:** 2026-03-20

**Authors:** Christiane Jungen, Christina Kohn, Jouny Koukash, Jan Bohnen, Dimitra Vlachopoulou, Christian Eitel, Leonie Kohlstadt, Sebastian Dorna, Hunar Haydar, Nele Riffelmann, Tienush Rassaf, Shibu Mathew

**Affiliations:** Department of Cardiology and Vascular Medicine, West German Heart and Vascular Center Essen, University of Duisburg-Essen, Essen, Germany

**Keywords:** Pulmonary vein isolation, Pulsed field ablation, Atrial fibrillation, Mapping catheter, Integrated mapping and ablation, Novel system

**Key Findings**

▪ Pulmonary vein isolation using an integrated mapping-ablation variable-loop pulsed field ablation catheter achieved high acute procedural success with a favorable safety profile.
▪ Procedural efficiency improved during early clinical adoption, with reductions in mapping and left atrial dwell times, reflecting a measurable learning curve.
▪ Left atrial anatomy influenced procedural workflow, with larger atria associated with longer mapping and dwell times.
▪ Anatomy-adapted catheter maneuvers, particularly for the right inferior pulmonary vein, facilitated successful ablation in challenging anatomies.

Pulmonary vein isolation (PVI) using pulsed field ablation (PFA) has recently emerged as a nonthermal, tissue-selective treatment of atrial fibrillation (AF) ablation with a favorable safety profile compared with thermal technologies.^1^ Novel systems integrating mapping and ablation within a variable-loop circular catheter (VLCC) may streamline workflow; however, real-world experience regarding procedural efficiency, anatomical influences, and catheter handling during early adoption remains limited.^2^ We report workflow performance and practical handling insights from the first consecutive patients treated with an integrated VLCC PFA platform.

Written informed consent was obtained from all patients before the procedure. The study adhered to the Declaration of Helsinki and was approved by the local ethics committee (registration number: 25-12749-BO). Pre-, intra-, and postprocedural management was performed as described previously.^3^ 50 consecutive patients with symptomatic AF underwent first-time PVI using a VLCC (VARIPULSE, Biosense Webster [Irvine, CA], Johnson & Johnson). Procedures were guided by integrated 3-dimensional electroanatomic mapping. *Major adverse events* were defined as death or complications requiring intervention or prolonging hospitalization beyond 48 hours.^4^

Patients (70% male, n = 35) were 67 ± 11 years old with 54% having paroxysmal AF, had a mean left ventricular ejection fraction of 57% ± 7%, and a mean left atrial volume index of 35 ± 14 mL/m^2^, with 54% having paroxysmal AF. Acute PVI was achieved in all patients, consistent with prior PFA experience.^1^ No major device-related complications occurred; 1 deep vein thrombosis prolonged hospitalization.

The mean procedure time was 73 ± 16 minutes and the left atrial dwell time was 46 ± 11 minutes, with 4352 ± 1908 mapping points acquired per case. Left atrial size influenced the procedural workflow. Left atrial volume index correlated with mapping time (R^2^ = 0.24; 95% confidence interval 0.24–0.68; *P* = .0003) and showed a trend toward a longer left atrial dwell time (R^2^ = 0.14; 95% confidence interval 0.11–0.59; *P* = .07). These findings suggest that atrial size affects catheter maneuverability and the extent of mapping required to achieve circumferential coverage, an observation consistent with anatomical influences described for other AF ablation technologies.^5^

A significant learning curve was observed. Left atrial dwell time decreased from 50 ± 11 minutes in the first 25 procedures to 41 ± 10 minutes in the last 25 (*P* = .007). Mapping time declined from 11 ± 6 to 7 ± 4 minutes (*P* = .002). Procedural experience correlated with reductions in left atrial dwell time (*R*^2^ = 0.177; *P* = .002) and mapping time (R^2^ = 0.239; *P* = .003), whereas total procedure duration remained unchanged (see Figure 1A for mapping before and after ablation). These improvements mainly reflected better catheter positioning strategies rather than global workflow changes, consistent with learning curve effects described for other PFA systems.^2^

**Figure 1:**
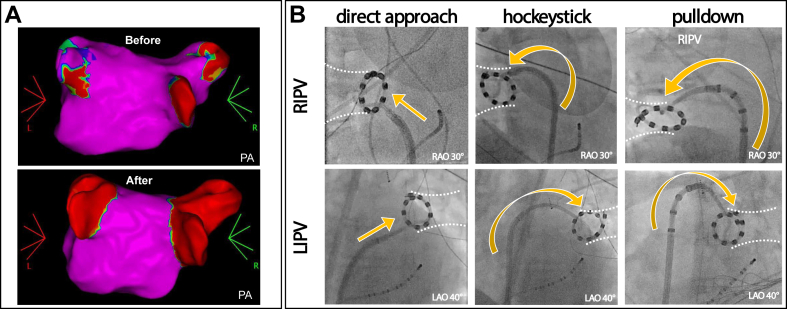
**A:** Mapping before and after pulsed field ablation applications. **B:** Different approaches for the right inferior pulmonary vein (RIPV) (upper panel) and left inferior pulmonary vein (LIPV) (lower panel). In addition to the direct approach (left), a hockey-stick (middle) or pull-down (right) maneuver is helpful in difficult anatomies. Arrows denote the direction of the maneuver. LAO = left anterior oblique; PA = posterior anterior; RAO = right anterior oblique.

The VLCC is stiffer than multipolar mapping catheters, requiring controlled sheath curvature and avoidance of maximal deflection. Excessive torque in highly flexed positions led to mechanical stress; in 2 cases, sheath damage necessitated exchange. Optimal performance was achieved in normal-sized or moderately enlarged atria. Very small atria restricted loop expansion and required careful incremental rotation rather than wide deflection. Larger atria demanded broader rotational sweeps to maintain tissue contact.

The right inferior pulmonary vein (RIPV) represented the most challenging target, particularly when originating low at the atrial floor. In 16% of patients (n = 8), a small RIPV or an RIPV located at the level of the floor of the left atrium was present, and so a hockey-stick maneuver was performed (Figure 1B). A “hockey-stick” sheath configuration or gentle pull-down maneuver improved alignment in these anatomies if a direct approach was not possible (Figure 1B). In oversized veins or common ostia, more antral positioning with gradual clockwise rotation around the ostium improved circumferential contact. These adjustments reduced the need for repeated repositioning as operator experience increased. Nevertheless, PVI remained feasible in all patients, underscoring the adaptability of the variable-loop design across a spectrum of atrial geometries.

Together, these observations indicate that procedural efficiency during early VLCC PFA adoption depends on both anatomical factors and familiarity with catheter mechanics. Understanding device stiffness, avoiding extreme sheath curvature, and applying adapted maneuvers in inferior or atypical veins were key contributors to improved mapping efficiency over time.

Limitations include the single-center design, modest sample size, and focus on acute procedural parameters without long-term rhythm follow-up. Nevertheless, these early workflow insights may assist centers adopting integrated mapping-ablation PFA systems.

In summary, early use of an integrated variable-loop PFA catheter demonstrates a measurable learning curve, with improvements in mapping and left atrial dwell efficiency driven by refined catheter handling. Left atrial anatomy significantly influences maneuverability and workflow, underscoring the importance of anatomy-adapted technique during early technology adoption.

## Disclosures

The authors have no conflicts of interest to disclose.
